# Liver Spots

**Published:** 1891-04

**Authors:** 


### LIVER SPOTS.

A writer in the *Boston Herald* has this to say of these provoking discolorations.

What are known as liver spots appear on both men and women, but they are commonly seen on the former, and very generally on those who are rapidly nearing or have passed middle life. These are spots of a yellowish brown color, and have quite sharply defined margins. In fact, they look like stains.

The forehead is the favorite seat of liver spots; but they appear elsewhere on the upper portion of the face. More often than otherwise the spots are small and few in number, and but slightly disfiguring. In occasional cases, however, a considerable area is involved, producing a very unsightly effect.

The actual cause of liver spots is an unusual accumulation of coloring matter in the skin; this results from some defect in the nerves which have the placing of this matter. Beyond this the essential causative conditions have never been uncovered. Doubtless there are a variety of derangements in different parts of the system capable of upsetting the nerves in question, and so causing the formation of liver spots ; but nothing is positively known on this point.

The removal of liver spots is always a difficult matter, and very rarely indeed can it be effected. There are many preparations on the market claimed to be positively efficacious, but none are so, and very few of them have any value whatever. Calomel and corrosive sublimate were once supposed to produce the most favorable results ; hence they became popular ingredients of complexion lotions, "beautifiers," etc. But these rarely have any noticeable effect upon liver spots.

Unless these spots are highly disfiguring it were better to leave them undisturbed. If treatment is applied it should be directed by a specialist in diseases of the skin. Before seeking one, the family physician should be consulted, for there are many charlatans who pretend to skill in this line of treatment.

